# Hypoxia promotes cell proliferation by modulating E2F1 in chicken pulmonary arterial smooth muscle cells

**DOI:** 10.1186/2049-1891-4-28

**Published:** 2013-07-31

**Authors:** Ying Yang, Feng Sun, Chen Zhang, Hao Wang, Guoyao Wu, Zhenlong Wu

**Affiliations:** 1 State Key Laboratory of Animal Nutrition, College of Animal Science and Technology, China Agricultural University, Beijing 100193, P.R. China; 2 Department of Pharmacy, National University of Singapore, 18 Science Drive 4, Singapore 117543, Singapore; 3 Department of Animal Science, Texas A&M University, College Station, TX 77843, USA; 4 Department of Animal Nutrition and Feed Science, College of Animal Science and Technology, China Agricultural University, Beijing 100193, P.R. China

**Keywords:** E2F1, Hypoxia, Proliferation, Pulmonary arterial smooth muscle cells

## Abstract

In this study, we sought to investigate the expression of the transcription factor E2F1 in chicken pulmonary arterial smooth muscle cells upon hypoxia exposure, as well as the role that E2F1 played in the regulation of cell proliferation. Isolated chicken pulmonary arterial smooth muscle cells were subjected to hypoxia or normoxia for indicated time points. Cell viability, DNA synthesis, cell cycle profile, and expression of E2F1 were analyzed. The results showed that hypoxia promoted cell proliferation and DNA synthesis which was accompanied by an increased S phase entry and upregulation of E2F1 at mRNA and protein levels. Using siRNA technology, we demonstrated that gene inactivation of endogenous E2F1 abolished hypoxia-induced cell proliferation, DNA synthesis, and S phase entry compared with negative siRNA transfected cells. These results suggest that hypoxia-induced proliferation is mediated by inducing E2F1 in chicken pulmonary arterial smooth muscle cells.

## Introduction

Pulmonary arterial hypertension is a disease of the small pulmonary arteries characterized by vascular dysfunction and structural remodeling [1,2]. Vascular remodeling leads to an increase of pulmonary vascular resistance, sustained arterial hypertension and ultimately results in right ventricular hypertrophy and death [3-5]. Both physical (for example, mechanical stretch, shear stress) and chemical (hypoxia, vasoactive substances, growth factors) stimuli have been reported to be risk factors associated with vascular remodeling in various animal models or humans. Among them, hypoxia plays an important role by modulating the production of anti-mitogenic factors, mitogenic factor, the release of inflammatory cytokines, and the induction of hypoxia-related transcription factors [4,6]. During the vascular remodeling process in response to stimuli, the predominant cell types (fibroblasts, smooth muscle cells, and endothelial cells) within each of the three layers of the blood vessel wall are affected. More and more recent evidence shows that the abnormal proliferation of smooth muscle cells might be the primary alteration that complicates and contributes to hypoxia-induced vascular remodeling [7].

Pulmonary arterial hypertension, also known as ascites syndrome in broilers, is one of the metabolic diseases characterized by an enlarged flaccid heart, variable liver changes, and accumulation of fluid in the abdominal cavity [8]. It is a widely used experimental model due to its apparent characteristics of pulmonary hypertension and vascular remodeling, as well as its highly reproducible in experimental condition [9,10]. Even though the etiology of ascites syndrome appears to be multi-factorial, it is generally believed that hypoxia promoted cellular proliferation due to the demand for oxygen exceeds its cardiopulmonary capacity is the predominant stimulus associated with the development of vascular remodeling [6,8,9,11]. We and others are interested in uncovering the underlying mechanisms and providing potential therapeutic strategies to alleviate the detrimental effect of ascites on broilers in the past few years [9,10,12,13]. In our recent study, we found that exposure to hypoxia induces pulmonary hypertension characterized by vascular remodeling and right ventricular hypertrophy in broilers which can be reduced by sodium hydrosulfide administration *in vivo*[14]. However, the underlying mechanisms are unknown.

E2F1 is a transcription factor with multiple cellular functions in regulating cellular response such as cell proliferation, differentiation, development, and apoptosis [15,16]. De-regulation of E2F1 has been reported to be associated with cell proliferation in many cell lines. In normal cells, the activity of E2F1 is tightly controlled to keep cellular homeostasis though multiple levels of regulation. In response to stress or mitogenic stimulus, E2F1 can promote cell cycle progression by activating downstream genes implicated in G1/S transition [17]. Also it has been reported that E2F1 can be induced in response to hypoxia in human pulmonary arterial smooth muscle cells [18]. These data suggest that E2F1 might be a candidate gene that regulating the proliferation of smooth muscle cells and contributes to hypoxia-induced vascular remodeling in broilers. Therefore, the aim of this study was to evaluate whether hypoxia-induced proliferation of chicken pulmonary arterial smooth muscle cells was mediated by E2F1 activation.

## Materials and methods

M199 and DMEM media, fetal bovine serum (FBS), antibiotics (penicillin and streptomycin), and other cell culture supplements were obtained from Invitrogen (Carlsbad, CA). Protease and phosphatase inhibitor cocktails were obtained from Roche Molecular Systems, Inc. (Alameda, CA). Molecular weight markers and reagents for protein determination were from Bio-Rad (Cambridge, MA). Antibodies against E2F1, CCNE1, β-actin, and horseradish peroxidase-conjugated goat anti-rabbit immunoglobulin G were from Santa Cruz Biotechnology (Santa Cruz, CA). Polyvinylidene difluoride (PVDF) membrane was obtained from Amersham Pharmacia Biotech. Other reagents used in this study were obtained from Sigma (St. Louis, MO.) otherwise as stated.

### Isolation and culture of chicken pulmonary arterial smooth muscle cells (PASMCs)

All animal care and procedures were in accordance with institutional and international guidelines. Twenty day-old chicken embryo pulmonary arterial smooth muscle cells (PASMCs) were isolated by enzymatic digestion as previously described [19,20]. In brief, proximal pulmonary arteries were isolated under aseptic condition and were cut into small pieces after removal of fat, adventitia, and connective tissue surrounding the arteries. After incubation for 90 min in DMEM medium supplemented with elastase type III (0.125 mg/mL), collagenase type I (0.5 mg/mL), and antibiotics (100 U/mL penicillin and 100 μg/mL streptomycin), the tissues suspension was centrifuged for 15 min at 1,500 g and the pellet was resuspended in M199 media supplemented with 10% FBS, 100 U/mL penicillin, 100 μg/mL streptomycin, 8 mmol/L HEPES, and 2 mmol/L glutamine at 39°C in a humidified atmosphere of 5% CO2/95% air environment. PASMCs were passaged with 0.05% trypsin and cells of passages 3–8 were used in all the experiments. 95% percent cells exhibited specific immunostaining by anti-α-smooth muscle-actin antibody, a generally accepted marker of smooth muscle cells [21] as expected.

### Cell proliferation assay

Proliferation of PASMCs was measured by 3-(4, 5-dimethylthiazol-2-yl)-2, 5-diphenyl tetrazolium bromide (MTT) assay as previously described [18]. Briefly, cells were seeded in 96-well plates at a density of 2,000 cells each well under normoxic or hypoxic conditions for indicated time points. At the end of treatment, 10 μL MTT (5 mg/mL)/well was added to each well of the plates, and incubated for another 4 h at 37°C. The supernatant was then carefully removed, and 75 μL/well dimethyl sulfoxide (DMSO) was added to dissolve the formazan crystals. The absorbance of the solubilized product at 570 nm was measured with microplate spectrophotometer (PowerWave XS, BioTek Inc, Vermont).

### [^3^H] Thymidine incorporation assay

Thymidine incorporation assay was performed according to the method described previously [20,22]. Briefly, PASMCs were seeded in complete growth medium at a density of 20,000 cells/well in 24-well plates. After 24 h, cells were growth-arrested by serum starvation with low serum medium (0.1% FBS) for 48 h. Arrested cells were labeled with [^3^H]-thymidine at 25Ci/mL and then were subjected to normoxia or hypoxia. Cells were harvested at indicated time points in 1% sodium dodecyl sulfate (SDS)/0.01 mol/L NaOH. [^3^H] thymidine incorporation was determined in a Becton scintillation counter (model LS6500, Franklin Lakes, NJ).

### Cell Cycle Analysis

Cell cycle was determined by flow cytometry as described previously [23]. Treated cells were harvested and washed two times with cold PBS, and then were fixed with 1 mL of 70% ethanol overnight at 4°C. Fixed cells were centrifuged for 3 min at 1,200 g and then were washed with cold PBS and resuspended in PBS with 50 mg/mL prepodium iodide (PI) and 1 mg/mL RNase. The stained cells were analyzed for DNA content by fluorescence-activated cell sorting (FACs) in a FACs Calibur (Becton Dickinson Instrument, San Jose, CA). Cell cycle fractions were quantified using the CellQuest software (Becton Dickinson).

### Quantitative real-time PCR

Total RNA was extracted from the PASMCs using an RNeasy Mini kit (Qiagen, Valencia, CA) according to the manufacturer's instructions and estimated using optical density measurements. Total RNA (2 μg) was used for first-strand cDNA synthesis using random hexamer primers and Superscript Reverse Transcriptase (Invitrogen, Carlsbad, CA). Quantitative real-time PCR was used to quantify the expression level of E2F1. PCR reactions were set up with SYBR Green PCR Supermix (Bio-Rad, Hercules, CA) using 1 μL of cDNA in each 20-μL reaction mixture). The relative values of gene expression were calculated using the ΔΔC_T_ method with β-actin as the internal control. The values were normalized to the average value of the control samples.

### siRNA transfection

Transfection reagent Lipofectamine-2000 was obtained from Invitrogen (Carlsbad, CA). The procedure for siRNA transfection was performed as previously described [18]. 48 h after siRNA transfection, cells were seeded in 6-well plates and subjected to normoxia or hypoxia for another 24 h; cells were then collected for Western blot analysis or DNA synthesis assay.

### Western blot analysis

The total lysate was obtained from PASMCs using ice-cold lysis buffer containing 20 mmol/L Tris–HCl (pH 7.4), 2.5 mmol/L EDTA, 1% Triton X-100, 1% sodium deoxycholate, 0.1% SDS, 200 mmol/L NaF, 100 mmol/L Na_3_VO_4_ and 1 mmol/L protease inhibitor cocktail from Roche (Alameda, CA). Cell lysates were centrifuged for 15 min at 16,000 g to remove cellular debris. Equal amounts of protein were separated on SDS-page gels and transfer to PVDF membranes (Millpore, Bedford, MA). The membranes were blocked in 5% non-fat milk at room temperature for 1 h, and then were incubated with primary antibodies against β-actin, E2F1, CCNE1, overnight at 4°C and then washed three times with PBST. The membranes were incubated with horseradish peroxidase-conjugated secondary antibody for 1 h. The signal detection was performed by using the enhanced chemiluminescence substrates (ECL Plus reagent; Amersham Pharmacia Biotech, San Diego, CA).

### Statistical analysis

Comparisons between groups were performed using two-way ANOVA followed by the Duncans test. Differences were considered statistically significant at the level of *P* <0.05 and values are represented as means ± SEM. The statistical analysis was performed with the software of SPSS 11.0 for Windows.

## Results

### Effect of hypoxia on the proliferation and DNA synthesis in chicken PASMCs

**Figure 1:**
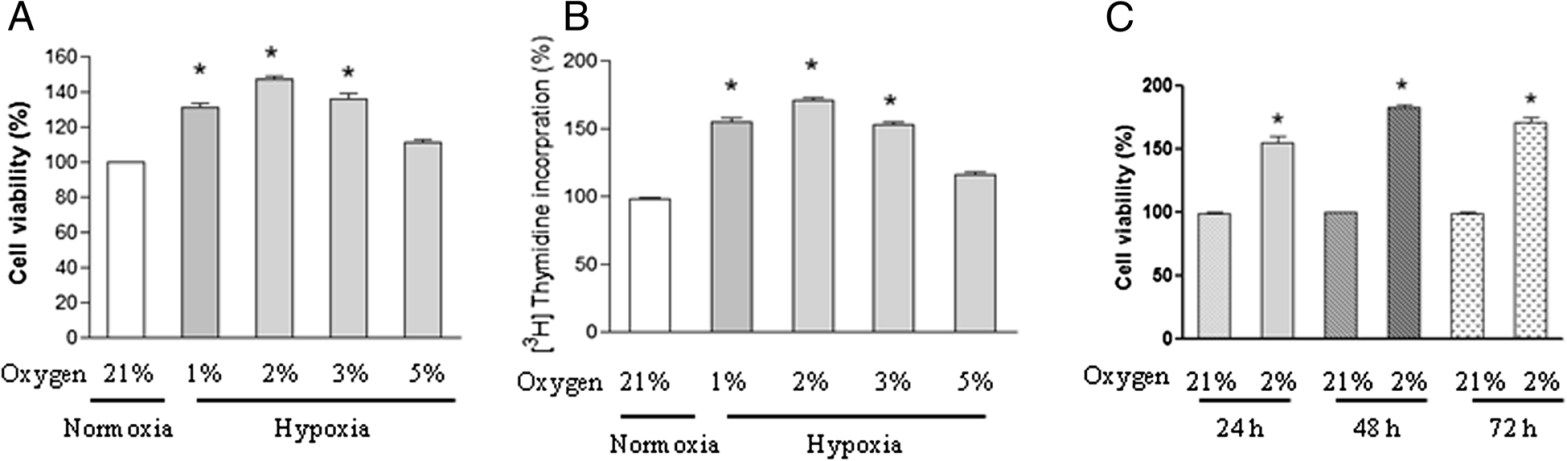
**Effect of hypoxia on the proliferation of chicken pulmonary arterial smooth muscle cells (PASMCs).** PASMCs were exposed to normoxia (21% oxygen) or under different concentrations of hypoxia (1%, 2%, 3%, or 5% oxygen) for 24 h, cell viability **(A)** and DNA synthesis **(B)** were analyzed by using MTT assay and [^3^H]-thymidine incorporation assay respectively. Each value is mean ± SEM of five separate experiments, each performed in triplicate. **(C)** PASMCs were exposed to normoxia (21% oxygen) or hypoxia (2% oxygen) for indicated time points, cell viability was determined and expressed as mean ± SEM of five separate experiments, each performed in triplicate. Values for the normoxic cells at each time point were set as 100%, *P<0.05 compared with normoxic control.

Arrested PASMCs were subjected to normoxic (21% oxygen) or different oxygen as indicated (1%, 2%, 3%, or 5%) for 24 h and cell proliferation was analyzed. A significant increase in cell viability was observed in PASMCs exposed to 1%, 2%, and 3% oxygen as evidenced by MTT assay (Figure 1A) and [^3^H]thymidine incorporation assay (Figure 1B). 5% oxygen induced a modest proliferation in PASMCs, and the difference was not significant. Moreover, the pro-proliferative effect of hypoxia (2% oxygen) on the proliferation of PASMCs was time-dependent and sustained to 72 h compared with normoxic control cells at each time point (Figure 1C). Because 2% oxygen exposure has the greatest effect on cell proliferation, we choose it for further study.

### Hypoxia-induced cell cycle progression is associated with activation of E2F1 in chicken PASMCs

**Figure 2:**
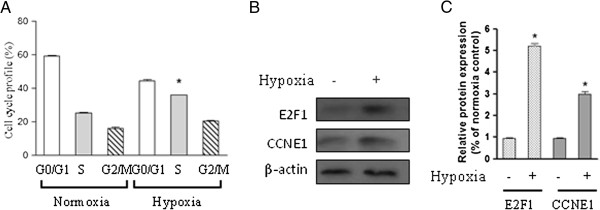
**Hypoxia induces S phase entry and E2F1 activation in chicken PASMCs.** Chicken PASMCs were cultured under normoxic (21% oxygen) or hypoxic (2% oxygen) conditions for 24 h. **(A)** Cell cycle profile was determined using the method as described in materials and methods. **(B)** Cells were treated as in **(A)**, and the protein level of E2F1, CCNE1, and β-actin were analyzed. **(C)** The relative expression levels of E2F1 and CCNE1 were determined from the immunoblots by densitometric analysis. *Values are mean ± SEM (n=5)*. *P<0.05 compared with normoxic control.

Because the proliferation of PASMCs was markedly promoted following hypoxia exposure (2% oxygen), we next determined whether the cell cycle was also changed in response to hypoxia exposure. Flow cytometry analysis showed that hypoxia promoted cell cycle progression as shown by an increased cell population in S phase compared with control cells cultured under normoxic condition (Figure 2A). To determine whether the transcription factor E2F1 was involved in the S phase entry observed, protein was extracted from cells in the presence or absence of hypoxia for 24 h. Western blot results showed that hypoxia significantly up-regulated E2F1 protein level as well as downstream target CCNE1 (Figure 2B and C). This result indicates that hypoxia-induced G1/S transition is associated with induction of E2F1 in our system.

### Silencing of E2F1 reduced hypoxia-induced cell proliferation and DNA synthesis

**Figure 3:**
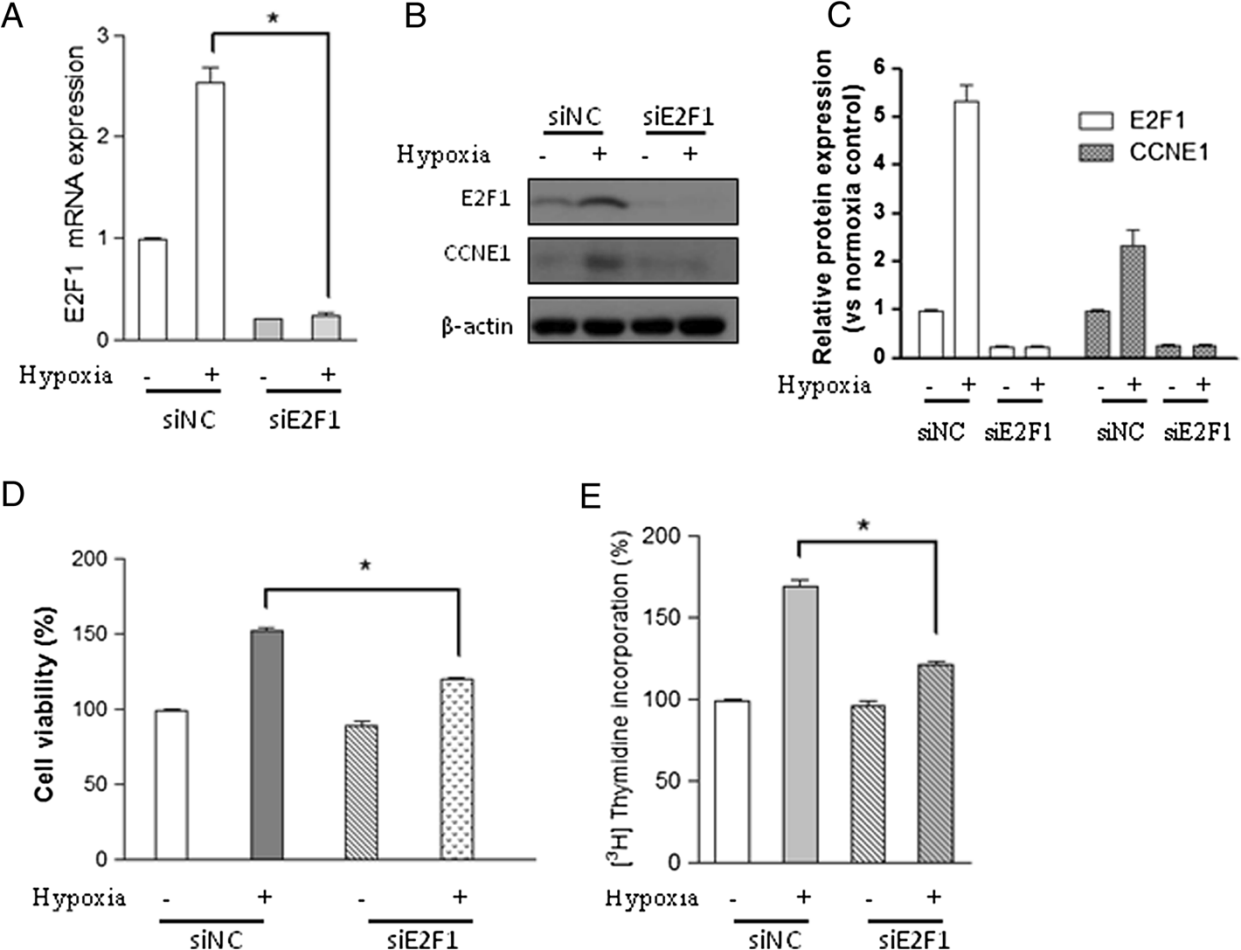
**E2F1 inactivation abolishes hypoxia-induced proliferation and DNA synthesis in PASMCs.** Cells transfected with negative control siRNA (siNC) or E2F1 siRNA (siE2F1) were cultured under hypoxic (2% oxygen) or normoxic conditions for 24 h. **(A)** The mRNA level of E2F1, **(B)** the protein levels of E2F1, CCNE1, and β-actin, **(C)** the relative protein levels of E2F1 and CCNE1 were determined from the immunoblots by densitometric analysis. **(D)** Cell viability and **(E)** DNA synthesis were determined using the methods as described in materials and methods. Values are mean ± SEM (n=5). *P<0.05 compared with hypoxic cell transfected with negative siRNA.

To test whether the E2F1 signaling pathway is responsible for hypoxia-induced cell proliferation and DNA synthesis, Chicken PASMCs transfected with negative siRNA or E2F1 specific siRNA were subjected to normoxia (21% oxygen) or hypoxia (2% oxygen). Real time-PCR (RT-PCR) result showed that endogenous E2F1 mRNA level was greatly reduced by E2F1 siRNA compared with control cells (siNC) (Figure 3A). Moreover, hypoxia-induced E2F1 up-regulation was markedly reduced by E2F1 siRNA, but not by negative control siRNA (Figure 3A). This result was validated by Western blot analysis (Figure 3B and C). MTT and DNA synthesis assays demonstrated that hypoxia-induced cell proliferation and DNA synthesis were significantly blocked in siE2F1 transfected cells, but not in the control cells (Figure 3D and E), indicating that E2F1 is responsible for hypoxia-induced cell proliferation effect in chicken PASMCs.

## Discussion

The key finding of this study is hypoxia-induced cell proliferation is associated with up-regulation of the transcription factor E2F1 in chicken pulmonary arterial smooth muscle cells (PASMCs). Gene inactivation of E2F1 using siRNA demonstrated that up-regulation of E2F1 is responsible for the proliferative effect observed in our system.

The control of cellular proliferation, including the entry from quiescence (G0) into the cell cycle (G1) and passage into the DNA replication (S) phase, is tightly regulated by multiple regulators to maintain cellular homeostasis. The E2F1 transcription factor has been well known for its ability to regulate cell cycle progression by controlling the expression of genes essential for the entry into the S phase of the cell cycle, such as CCNE1 (Cyclin E1) and CCNA2 (Cyclin A2) [24-26]. The retinoblastoma (Rb) protein is a negative regulator of E2F1 and is controlled by the activity of upstream cyclin-dependent kinases (CDKs) that are activated during the transition from G0 to G1. Cyclin-dependent kinase inhibitors, or CKIs, can block activation of CDKs and thus regulates the activity and related function of E2F1. Due to the existence of negative and positive regulators, the cellular proliferation activity of E2F1 is well controlled. Disruption of components of this pathway leads to deregulated proliferation [15]. In this study, we, for the first time, demonstrated that hypoxia exposure up-regulates E2F1 as well as CCNE1, a downstream target that associated with G1/S transition [27] in chicken PASMCs. As expected, activation of E2F1 was accompanied by an increase of S phase cell population and DNA synthesis. Interestingly, hypoxia-induced phenotype was abolished by E2F1 siRNA transfection, supporting a critical role of E2F1 in this stress response. This result was in agreement with a recent study showing that repressing E2F1 reversed hypoxia-induced proliferation in mice PASMCs [18]. More study is needed to elucidate the precise mechanism that responsible for the proliferative effect observed upon hypoxia exposure. It should be noted that besides E2F1, E2F2 and E2F3 can also activate genes associated with G1/S transition and involve in cell cycle progression [15,16]. Further study is needed to clarify whether E2F2 or E2F3 is activated in response to hypoxia exposure and contribute to this effect.

Hypoxia-inducible transcription factors (HIFs), including HIF-1α, HIF-2α, and HIF-3α, were reported to be expressed in hypoxic smooth muscle cells and regulate gene expression via the hypoxia-responsive-element [28]. Studies of mice partially deficient for HIF-1α showed delayed development of pulmonary vascular remodeling, pulmonary hypertension, and right ventricular hypertrophy [29], suggesting an important role of HIF-1α in the development of vascular remodeling. We did not determine the expression level of HIF-1α due to the unavailability of antibody that cross-react with chicken PASMCs. Considering the diverse downstream targets of HIF-1α and E2F1, It will be of great significance to investigate the potential interaction between these two signaling pathways.

In conclusion, we demonstrated that hypoxia exposure promotes proliferation in chicken pulmonary arterial smooth muscle cells by activation of transcription factor E2F1 which can be blocked by siRNA-mediated gene silence. This observation revealed a novel mechanism underlying the regulation of proliferation by E2F1 in hypoxic pulmonary hypertension and vascular remodeling. Blocking E2F1 activity through genetic modification or small molecule might be a potential strategy for the inhibition of hypoxia-induced pulmonary vascular remodeling.

## Competing interests

The authors declare that they have no competing interests.

## Authors’ contributions

Ying Yang conducted the cell culture, cell proliferation assay and siRNA transfection. Feng Sun participated in the design of the experiments and performed Real-time PCR. Ying Yang did the Western blot, and Chen Zhang did the cell cycle profile analysis. Guoyao Wu and Zhenglong Wu conceived the experiment and conducted the statistical analysis. All authors participated in writing the manuscript and have read and approved the final manuscript.
